# IL-37 exerts therapeutic effects in experimental autoimmune encephalomyelitis through the receptor complex IL-1R5/IL-1R8

**DOI:** 10.7150/thno.47435

**Published:** 2021-01-01

**Authors:** Alba Sánchez-Fernández, Stephanie Zandee, Jesús Amo-Aparicio, Marc Charabati, Alexandre Prat, Cecilia Garlanda, Elan Z. Eisenmesser, Charles A. Dinarello, Rubèn López-Vales

**Affiliations:** 1 Institut de Neurociencies and Departament de Biologia Cel·lular, Fisiologia i Immunologia, Universitat Autonoma de Barcelona, Bellaterra, Catalonia, Spain.; 2 Centro de Investigación Biomédica en Red de Enfermedades Neurodegenerativas (CIBERNED).; 3 Department of Neuroscience, Faculty of Medicine, Université de Montréal, and Neuroimmunology Unit, Centre de Recherche du CHUM (CRCHUM), Montréal, Québec, Canada.; 4 Humanitas Clinical and Research Center, 20089 Rozzano, Italy.; 5 Department of Biochemistry and Molecular Genetics, University of Colorado Denver, Aurora, CO 80238, USA.; 6 Department of Medicine, University of Colorado Denver, Aurora, CO 80045, USA.; 7 Department of Medicine, Radboud University Medical Center, 6500 Nijmegen, The Netherlands.

**Keywords:** multiple sclerosis, experimental autoimmune encephalomyelitis, IL-37, IL-1R8, cytokines

## Abstract

**Background:** Interleukin 37 (IL-37), a member of IL-1 family, broadly suppresses inflammation in many pathological conditions by acting as a dual-function cytokine in that IL-37 signals via the extracellular receptor complex IL1-R5/IL-1R8, but it can also translocate to the nucleus. However, whether IL-37 exerts beneficial actions in neuroinflammatory diseases, such as multiple sclerosis, remains to be elucidated. Thus, the goals of the present study were to evaluate the therapeutic effects of IL-37 in a mouse model of multiple sclerosis, and if so, whether this is mediated via the extracellular receptor complex IL-1R5/IL-1R8.

**Methods:** We used a murine model of MS, the experimental autoimmune encephalomyelitis (EAE). We induced EAE in three different single and double transgenic mice (hIL-37tg, IL-1R8 KO, hIL-37tg-IL-1R8 KO) and wild type littermates. We also induced EAE in C57Bl/6 mice and treated them with various forms of recombinant human IL-37 protein. Functional and histological techniques were used to assess locomotor deficits and demyelination. Luminex and flow cytometry analysis were done to assess the protein levels of pro-inflammatory cytokines and different immune cell populations, respectively. qPCRs were done to assess the expression of IL-37, IL-1R5 and IL-1R8 in the spinal cord of EAE, and in blood peripheral mononuclear cells and brain tissue samples of MS patients.

**Results:** We demonstrate that IL-37 reduces inflammation and protects against neurological deficits and myelin loss in EAE mice by acting via IL1-R5/IL1-R8. We also reveal that administration of recombinant human IL-37 exerts therapeutic actions in EAE mice. We finally show that IL-37 transcripts are not up-regulated in peripheral blood mononuclear cells and in brain lesions of MS patients, despite the IL-1R5/IL-1R8 receptor complex is expressed.

**Conclusions:** This study presents novel data indicating that IL-37 exerts therapeutic effects in EAE by acting through the extracellular receptor complex IL-1R5/IL-1R8, and that this protective physiological mechanism is defective in MS individuals. IL-37 may therefore represent a novel therapeutic avenue for the treatment of MS with great promising potential.

## Introduction

Multiple sclerosis (MS) is the most common demyelinating disease of the central nervous system (CNS) and affects more than 2.5 million individuals worldwide 1. Although its aetiology remains complex, MS is thought to be autoimmune in nature due to the presence of myelin-targeting T cells and autoantibodies in the cerebrospinal fluid of MS patients 2-5. Therefore, blocking inflammation will likely be therapeutically beneficial for treating MS patients.

The IL-1 family includes prototypic cytokines that promote acute and chronic inflammation in a broad spectrum of diseases 6, 7. Seven of the 11 members exhibit pro-inflammatory properties, whereas four members reduce inflammation 8. Of these four, IL-37 broadly suppresses innate as well as adaptive immunity 8-10.

The IL-37 gene is composed by 6 exons that give rise to five different isoforms (IL-37a to IL-37e) 11, 12. However, only the isoforms *a, b* and* d* are functional since, contrary to the isoforms *c* and* e*, they have the presence of the complete sequence encoding the 12β-strands structure and at least one cleavage site required for the maturation of the cytokine 13. Among them, IL-37b (isoform 1) is the longer isoform and it is the one that has been used in experimental studies for its functional characterization 11.

IL-37 exert anti-inflammatory actions by acting as a ligand for the extracellular receptor complex comprised of IL‑1R5 (IL-18Rα) and the co-receptor, IL‑1R8 14. Similar to IL‑1α and IL‑33, intracellular IL‑37 translocates to the nucleus via caspase-1 cleavage and binding to SMAD3 13, 15.

Unlike other members of the IL-1 family, the open read frame for the IL-37 homolog is lacking in mice. To investigate the role IL-37 in animal models of human diseases, it was thus necessary to generate a knock-in mouse expressing human *IL37b* (hIL-37tg) 1^6^. hIL-37tg mice have demonstrated resistance against many pathological conditions, such as systemic endotoxemia 9, colitis 17 and obesity-induced inflammation 18. In parallel, treatment with recombinant human IL-37 protein improved aspergillosis 19 and rheumatoid arthritis 19, 20 by exerting its anti-inflammatory properties.

Little is known about the role of IL-37 in the central nervous system. We previously reported that IL-37 exerts marked anti-inflammatory effects after spinal cord contusion injury in mice. Notably, hIL-37tg mice displayed significant resistance against functional deficits and demyelination after spinal cord injury (SCI) 21. A recent report reveals that IL-37 is reduced in blood leukocytes of multiple sclerosis (MS) 22, suggesting that this anti-inflammatory pathway might be defective in MS. However, whether IL-37 has similar therapeutic effects in neuroinflammatory conditions, such as MS, has not been addressed yet.

In the present study, we investigated the anti-inflammatory and therapeutic potential of IL-37 in experimental autoimmune encephalomyelitis (EAE), a mouse model of MS. We demonstrate that the transgenic expression of human IL-37 mediates broad anti-inflammatory effects in EAE and protects mice from the functional deficits and demyelination. Furthermore, we show that the beneficial effects of IL-37 in EAE are dependent on its extracellular receptor function. Relevant to the studies in mice, we discovered that this natural anti-inflammatory mechanism is deficient in blood and brain active lesions of MS patients. Lastly, we provide clear evidence that the administration of recombinant human IL-37 protein markedly reduces EAE pathogenesis. Altogether, our findings reveal that IL-37 could be employed as a new approach for the treatment of MS.

## Materials and Methods

### MS brain tissue samples

Human brain tissue was obtained from MS patients diagnosed with clinical and neuropathological MS diagnosis according to the revised 2010 McDonald's criteria 23. Tissue samples were collected from MS patients with full ethical approval (BH07.001) and informed consent as approved by the local ethics committee. Autopsy samples were cryopreserved and lesions classified using Luxol Fast Blue (LFB)/Haematoxylin & Eosin staining and Oil Red O staining as previously published 24, 25. Normal appearing white matter (NAWM) and active lesions representing two relapsing-remitting MS (RRMS) (one male and one female) and seven secondary-progressive MS (SPMS) (three males and four females) were used (Table S1). The median age at death was 50 years (range from 26 to 65 years). Regions of interest (active lesion or NAWM) for RNA isolation were manually dissected from 5-6 cryosections per block (50 μm width).

### MS and healthy donor PBMC samples

Blood samples were collected from the following subjects: three from patients with secondary progressive (SPMS) (one male and two females), and eight from patients with relapsing-remitting MS (RRMS) (three males and eight females) (Table S1). All patients were recruited at the MS Clinic at the Research Center of the University of Montreal Hospital Center (CRCHUM). Their ages ranged from 27 to 65 years (mean age, 43 years). At time of blood sampling, only two patients were receiving treatment with disease modifying drugs (Table S1). Seven healthy volunteers were included as controls (four males and three females); their ages ranged from 24 to 42 years (mean age, 35 years). A written informed consent was obtained from patients and healthy donors in accordance with the local ethics committee (CRCHUM research ethic committee approval number BH07.001). PBMCs were isolated from blood samples collected in EDTA-coated vacutainer tubes (BD Biosciences, Oakville, ON, Canada) using a Ficoll density gradient (Amersham Biosciences) as described previously 26, 27.

### Mice

All experimental procedures were approved by the Universitat Autònoma de Barcelona Animal Experimentation Ethical Committee (CEEAH 2878) and followed the European Communities Council Directive 2010/63/EU, and the methods were carried out in accordance with the approved guidelines.

Experiments were performed in adult (8-10 weeks) female or male C57Bl/6 mice (Charles River), transgenic mice homozygote for human *IL-37b* (hIL-37tg), *Il1r8* deficient mice (*Il1r8* KO), hIL-37tg-Sigirr KO mice, and their respective wildtype littermates. Mice were randomly distributed in groups of 5-6 mice per cage. All mice were housed with food and water *ad libitum* at room temperature of 22±2ºC under 12:12 h light-dark cycle.

### EAE induction

Mice were sedated with intramuscular injection of mixture of ketamine (22 mg/kg) (Imalgen 1000, Merial) and xylazine (2.5 mg/kg) (Rompun, Bayer). EAE was actively induced by subcutaneously injection in each flank at the base of the tail of 100 µl of 3 mg/mL of myelin oligodendrocyte glycoprotein peptide 35-55 (MOG_35-55_) in Complete Freund's Adjuvant (CFA) (Difco, Detroit, MI, USA) supplemented with 4mg/mL of heat inactivated *Mycobacterium tuberculosis* (Difco, Detroit, MI, USA). Immediately after induction, and on day 2, mice received an intraperitoneal (i.p.) injection of 400 ng of pertussis toxin (PTX) in 100 μl sterile saline (0.9% NaCl).

### Functional evaluation of EAE mice

Mice were scored daily from day 0 to day 21 after induction of EAE. The researcher was blinded to experimental groups during the functional evaluation. A 6-point scale was used to evaluate the clinical signs of EAE: 0 = normal walking; 0.5 = partially paralyzed tail; 1 = fully paralyzed tail; 2 = mild hind limb weakness, quick righting reflex; 3 = severe hind limb weakness, slow righting reflex, unable to bear weight; 3.5 = severe hind limb weakness and partial paralysis of hind limb; 4 = complete paralysis of at least one hind limb; 4.5 = complete paralysis of one or both hind limbs and trunk weakness; 5 = complete paralysis of one or both hind limbs, forelimb weakness or paralysis; 6 = moribund. Functional data were pooled from two different experiments.

EAE onset was considered on the day animals showed the first signs of disease (EAE score 0, 5 or 1; around day 9-10 post-immunization). Disease peak was considered on the day EAE score did not increase from the previous day (around day 15-17 post-immunization). Chronic phase of the disease was considered at day 21 post-immunization if EAE score decreased or was maintained with respect to disease peak.

### Drug administration

Female C57Bl6/J mice were treated with daily intraperitoneal (i.p.) injections of 1 µg of native IL-37^46-218^ or the mutated monomeric forms IL-37^D73K^ or IL-37^Y85A^ in 200 µl of saline 28. Recombinant human IL-37 protein forms were administered when mice showed the first signs of the EAE disease, and then, daily until the end of the follow up. Control mice were treated with 200 µl of saline following the same protocol. Fingolimod was dissolved in distilled water and administered daily by oral gavage (0.3 mg/kg).

### Real-time Quantitative PCR Assay (qPCR)

Total RNA was isolated from post-mortem human brain active MS lesions and normal appearing white matter (NAWM), as well as, from spinal cords harvested of EAE mice harvested at different stages of the disease using the RNeasy Lipid Tissue Mini Kit (Qiagen) following the manufacturer's procedures. Total RNA was also extracted from PBMCs collected from MS patients and healthy donors using the QIAamp RNA Blood Tissue Mini Kit (Qiagen) according to the manufacturer's guidelines.

RNA was retrotranscribed using the cDNA Reverse Transcription Kit (Applied Biosystems). cDNA human libraries were analysed using a Bio-Rad CFX384 (CFX Manager V3.1) and TaqMan-designed primers (ThermoFisher Scientific) for the following human and mouse genes: *IL37b* (Hs00367201_m1), *IL1R8* (Hs00222347_m1), *IL1R5* (Hs00187256_m1), *GAPDH* (Hs02786624_g1), *Gapdh* (Mm03302249_g1), *Il1r8* (Mm01275624_g1), *Il1r5* (Mm00516053_m1). *GAPDH*/*Gapdh* was used as a housekeeping gene for human and mouse samples, respectively. All data were analysed using the 2^ΔΔ^Ct method. Data from EAE mice were pooled from two different experiments.

### Histological analysis

Female hIL-37tg mice and WT littermates were euthanised 21 days post EAE induction with an overdose of pentobarbital sodium (Dolethal) and transcardially perfused with 4% paraformaldehyde (PFA) in 0.1 M of phosphate buffer (PB). Lumbar segments of spinal cord were harvested, post-fixed in 4% PFA for 2 h on ice and cryoprotected in 30% sucrose in 0.1 M PB at 4ºC for at least 48 h. Next, spinal cords were embedded in Tissue-Tek^®^OTC (Sakura, Japan), sequential transversal sections (15 µm-thick) between L3 and L5 segments were cut using a Leica cryostat and gelatine-coated glass slides. Samples were stored at -20ºC until further use.

Spinal cord slides were stained with LFB (Sigma Aldrich). Briefly, after graded dehydration, sections were placed in 1 mg/mL of LFB solution in 96% ethanol and 0.05% acetic acid overnight at 37ºC and protected from light. Then, slides were washed with 96% ethanol, rehydrated with distilled water and differentiated using a 0.5 mg/mL Li_2_CO_3_ solution in distilled water for 3-5 min at room temperature. Finally, sections were washed in distilled water, dehydrated again and mounted in DPX mounting medium (Sigma Aldrich). To assess the demyelinated area in the spinal cords, 6 random images per spinal cord were captured at 10X magnification with an Olympus DP73 Camera attached to an Olympus BX51 microscope. The total demyelinated area in the spinal cord was measured with Image J image analysis software. Histological data were pooled from two different experiments.

### Cytokine protein expression assessment

Female hIL-37tg mice and WT littermates were euthanised 3 days after the onset of EAE with and overdose of pentobarbital sodium (Dolethal) and transcardially perfused with 60 mL of sterile saline (0.9% NaCl). Spinal cords were harvested and rapidly frozen using liquid nitrogen. To assess the protein levels of cytokines, spinal cord were processed as we previously published 29. Briefly, tissue was homogenized in HEPES and the protein concentration determined with a DC Protein Assay (Bio-Rad). Protein homogenates were concentrated to 4 µg/µl and finally, protein levels of IL-4, IL-10, IL-1α, IL-1β, IL-6, TNF-α, IFNγ, IL-17A, CSF-3, CCL-5, CCL-2, CXCL-2 and CXCL-10 were analysed using a custom-designed Mouse Cytokine Magnetic Bead Panel (Affymetrix, eBioscience) on a Luminex-MAGPIX system (Millipore). Luminex data were obtained from a single experiment.

### Flow cytometry

Female hIL-37tg mice and WT littermates were euthanised at peak of EAE with an overdose of pentobarbital sodium (Dolethal). 15 µl of blood was collected from a cardiac puncture and stored in heparinized vials at 4ºC. Then, mice were transcardially perfused with 60 mL of sterile saline (0.9% NaCl) and the spinal cords and lymph nodes (cervical and inguinal) were collected.

To analyse the different immune cells, the spinal cord, blood and lymph nodes were processed as we previously described in 29. Blood samples were incubated with red blood cell lysis buffer (BioLegend) according to manufacturer' guidelines to obtain a cell suspension enriched in leukocytes. Spinal cord and lymph nodes were cut in small pieces and enzymatically dissociated incubating them 30 min at 37ºC in 1 mL of HBSS without Ca^2+^/Mg^2+^ and 0.1% collagenase and 0.1% DNase. Next, samples were mechanically disintegrated by passing them with DMEM-10% FBS through a 70 µm cell strainer. After two washes, cell suspension was split into different 1.5 mL microcentrifuge tubes according to the number of antibody combinations. For surface staining, samples were incubated with the following primary antibodies from eBioscience for 1 h at 4ºC in soft agitation at a 1:300 concentration: CD45-PerCP, CD11b-PE or PE-Cy7, F4/80-PE or -APC, Ly6C-FITC, Ly6G-PE, CD3-FITC-APC-PerCP; CD4-APC-Cy7, CD8-APC, CD49b-PE, CD24-PE and the isotype matched-controls (1:300 eBioscience). Samples were then fixed with 1% PFA. When needed, intracellular staining was done after extracellular staining and cell fixation. For this purpose, cells were first permeabilizated with Permeabilization Wash Buffer (Biolegend) or FoxP3 Transcription Factor Staining Buffer Set (eBioscience) for 30 min in case of doing FoxP3 staining. Cell were then incubated for 45 min at 4ºC with FoxP3-PE-Cy7, tBet-PerCP, RORγ-APC, GATA3-PE, or the isotype matched-controls (1:300 eBioscience) or unconjugated rabbit antibody against iNOS (1:100 Abcam) and unconjugated goat antibodies against Arg1 (1:100 Santa Cruz) followed by staining with Alexa488 or Alexa647 conjugated donkey secondary antibodies against rabbit or goat (1:500 Molecular Probes) or isotype matched-control for 45 min. Samples were finally analysed on a FACS Canto Flow Cytometer (BD Bioscience) and all data were processed using FlowJo® software V.10. Flow cytometry data were obtained from a single experiment.

### Haematopoiesis assay

Bone marrow cells were obtained from femurs and tibia of WT and hIL-37tg mice after euthanasia. Cells were flushed out using a 21G-needle syringe with 5 ml of RPMI 1640 medium (ThermoFisher Scientific) supplemented with Penicillin/Streptomycin (Sigma-Aldrich). Cells were passed through a 70 μm cell strainer (FisherBrand). Counts of monocytes, lymphocytes, and granulocytes were determined using a HemaTrue cell counter (Heska, Loveland, CO, USA). Before measuring, cell counter machine was calibrated using a certified blood control authorized by Heska. At least 3 measures of the same sample were performed, and average values were taken.

### Experimental design and statistical analyses

All the experimental groups were gender- and age-matched. For all experiments, the number of mice used are stated in the figure legends and text. Data are shown as mean ± standard error of the mean (SEM). Statistical analysis was performed using GraphPad Prism 8. The distribution of the data was evaluated for normality using D'Agostino and Pearson test. Two tailed Student's *t* test was used for the comparison between two different groups (human qPCR, histological analysis, cytokine levels, accumulation of immune cells). Mean clinical score follow-up was analysed by using two-way ANOVA repeated measures with *post-hoc* Bonferroni's test for multiple comparisons. Differences were considered significant at *p*<0.05.

## Results

### Transgenic expression of *IL37* reduces neurological deficits and protects against demyelination in the spinal cord of EAE mice

**Figure 1:**
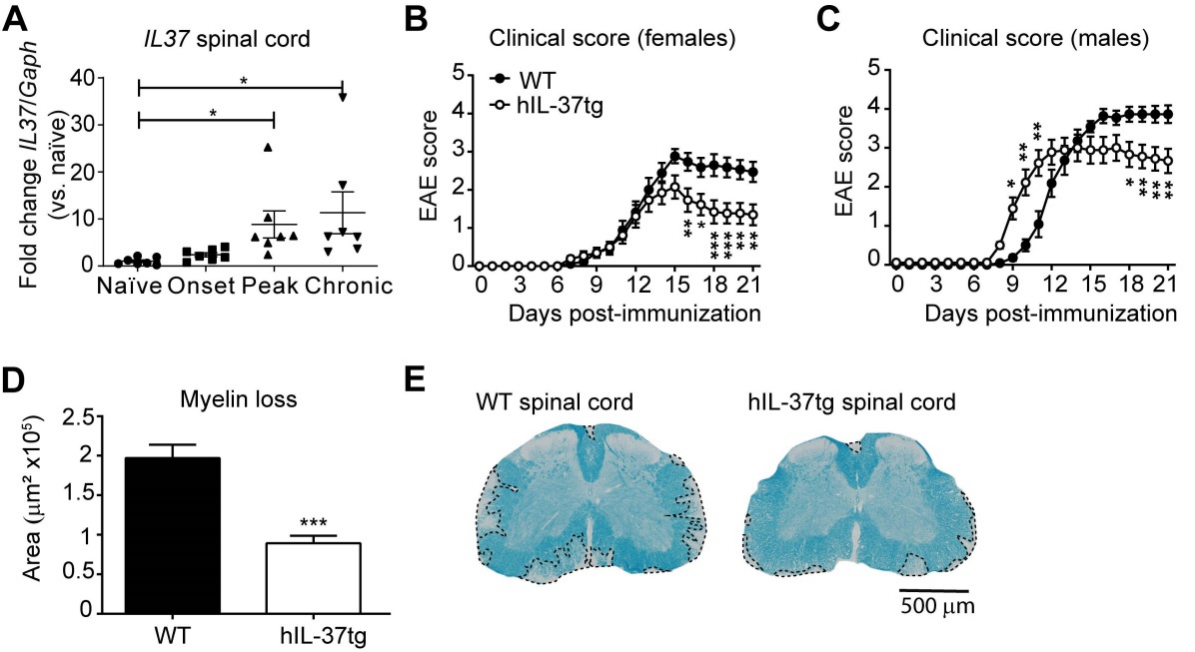
** The transgenic expression of *IL37* protects against functional deficits and myelin loss in the spinal cord of mice with EAE.** (**A**) Graph showing the levels of *IL37* mRNA in the spinal cord of female hIL-37tg mice at different phase of EAE disease. (**B**) Clinical score of females and (**C**) male hIL-37tg mice and WT littermates. (**D**) Graph showing the quantification of the area of demyelination in the spinal cord of female hIL-37tg and WT littermates at 21 days post induction. (**E**) Representative images of lumbar spinal cord from female hIL-37tg mice and WT littermates at day 21 after EAE induction. *p<0.05; **p<0.01; ***p<0.001 vs. control. One-way ANOVA with Bonferroni *post hoc* test in A (n=7 per time point); Two-way repeated measures ANOVA with Bonferroni's *post hoc* test in B (n=15 WT; n=14 hIL-37tg) and C (n=9 per group); Unpaired t-test in D (n=15 WT; n=14 hIL-37tg. Data were pooled from two different experiments. Data shown as mean±sem.

Since the role of IL-37 in demyelinating diseases of the CNS is still poorly understood, we first sought to explore whether this cytokine exerted beneficial effects in EAE mice. Since the open frame of the *IL37* orthologue is not present in mice, we induced EAE in hIL-37tg mice and wildtype littermates (WT). We found that *IL37* transcripts were detected at very low levels in the spinal cord of hIL-37tg at physiological conditions, but they were significantly increased (~7 fold) at the peak and chronic phase of the disease, but not at its onset (Figure 1A).

Knowing that the expression of *IL37* was inducible in the spinal cord upon EAE induction, we next evaluated its contribution to disease. Female hIL-37tg mice were significantly protected against functional deficits compared to WT mice (Figure 1B). At the end of the follow up, hIL-37tg female mice showed a ~1.5 point reduction in EAE clinical score. Similar beneficial effects were observed in male hIL-37tg mice suffering from EAE. However, the onset of disease occurred significantly earlier in male mice expressing *IL37* (Figure 1C).

We then evaluated whether the enhancement in functional outcomes of female hIL-37tg mice after EAE induction was linked to myelin preservation. Histological analysis of lumbar spinal cords harvested at the end of the follow up (21 days post induction) revealed that the area of demyelination was indeed decreased ~50% in the hIL-37tg mice (Figure 1D-E).

### IL-37 attenuates inflammation in mice with EAE

**Figure 2:**
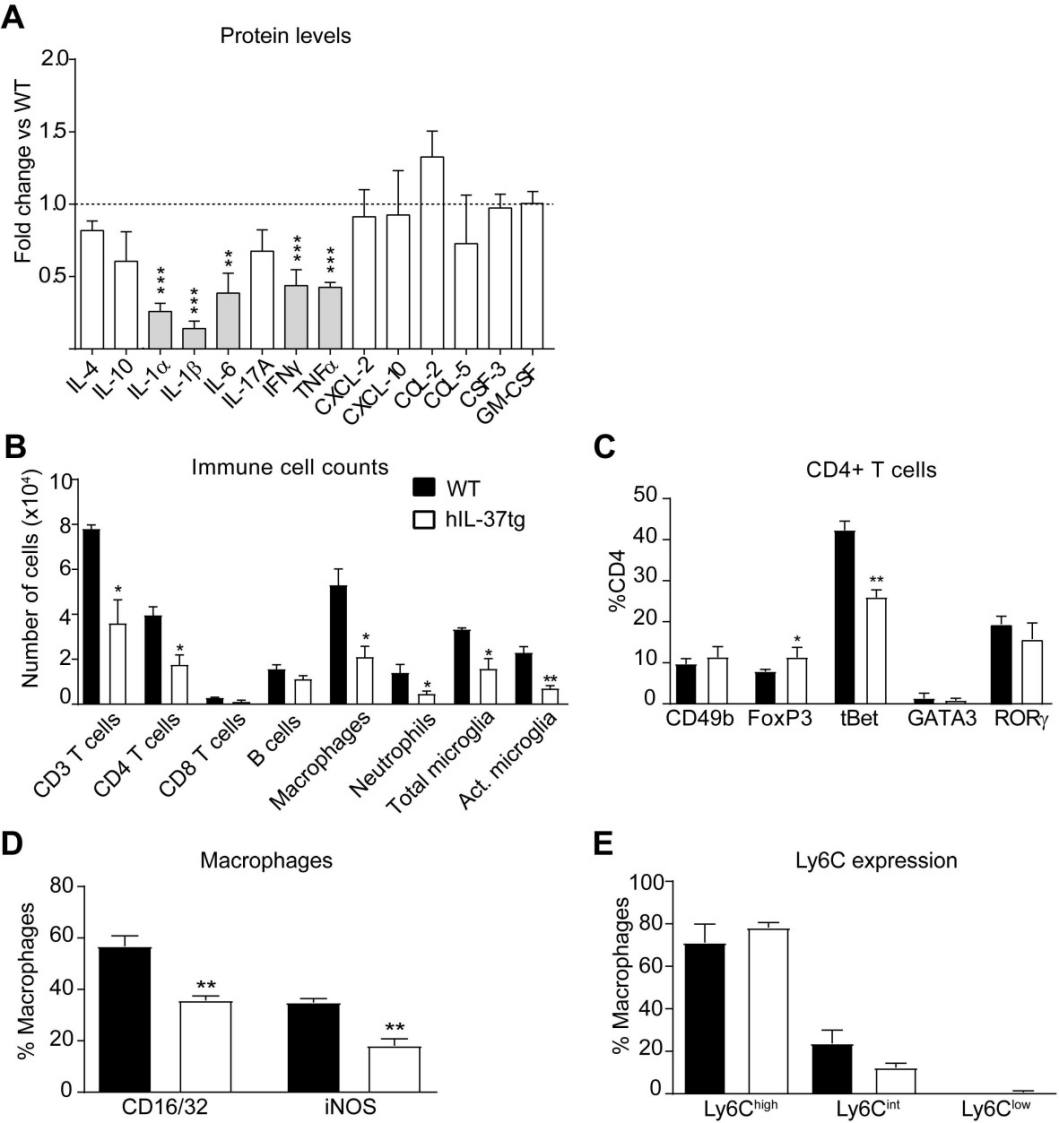
** IL-37 reduces the inflammatory response in the spinal cord of mice at the peak of EAE.** (**A**) Protein level profile of cytokines (IL-4, IL-10, IL-1α, IL-1β, IL-6, IL-17A, IFNγ, TNFα and chemokines (CXCL-2, CXCL-10, CCL-2, CCL-5, CSF-3) in the spinal cord of female hIL-37tg relative to female WT at the peak EAE. (**B**) Number of infiltrated immune cells and (**C**) percentage of CD4+ T cells expressing the transcription factors CD49b, FoxP3, tBet, GATA3 or RORγ in the spinal cord of female WT and female hIL-37tg mice at the peak of EAE. (**D**) Percentage of macrophages expressing the pro-inflammatory markers CD16/32 and iNOS or (**E**) Ly6C in the spinal cord of female WT and female hIL-37tg mice at the peak of EAE. *p<0.05; **p<0.01; ***p<0.001 vs. WT. Unpaired *t*-test (n=5 per group in A; n=4 per group in B-E). Data were obtained from a single experiment. Data shown as mean±SEM.

We next studied the anti-inflammatory actions of IL-37 in EAE. For this purpose, we first measured the protein levels of several cytokines in the spinal cord at day 3 after disease onset. A Luminex assay revealed that transgenic expression of IL-37 significantly reduced the protein levels of 5 pro-inflammatory cytokines (IL-1α, IL-1β, IL-6, TNFα and IFNγ), each of them linked to development of EAE pathophysiology (Figure 2A). Since cytokines coordinate the recruitment and activation of immune cells into the CNS parenchyma, we next evaluated whether transgenic expression of IL-37 reduced the accumulation of immune cells in the spinal cord at the peak of the disease. Flow cytometry analysis showed that hIL-37tg mice had significant lower numbers of T cells (CD3+), including CD4 and CD8 T cells, macrophages (CD45high, CD11b+, F4/80+), total microglial cells (CD45low, CD11b+) and activated microglial cells (CD45low, CD11b+, F4/80+) (Figure 2B, Figure S1-S2). IL-37, however, did not attenuate the number of B cells (CD45+, CD11b-, CD3-, CD24+) and neutrophils (CD45+, CD11b+, F4/80-, Ly6G+) (Figure 2B, Figure S2B, F). Of note, transgenic expression of *IL37* significantly reduced the proportion of pathogenic Th1 cells (CD3+, CD4+, tBet+) and increased the proportion of classical regulatory CD4 cells (CD3+, CD4+, FoxP3+) in the spinal cord (Figure 2C, Figure S3A). Moreover, IL-37 reduced the expression of CD16/32+ and iNOS+ in macrophages by 20% (Figure 2D, Figure S3B), but did not alter the expression of Ly6C (Figure 2E, Figure S3C). These data indicate that IL-37 not only reduced the accumulation of immune cells in the spinal cord, but also modulated their phenotype towards a more anti-inflammatory state.

**Figure 3:**
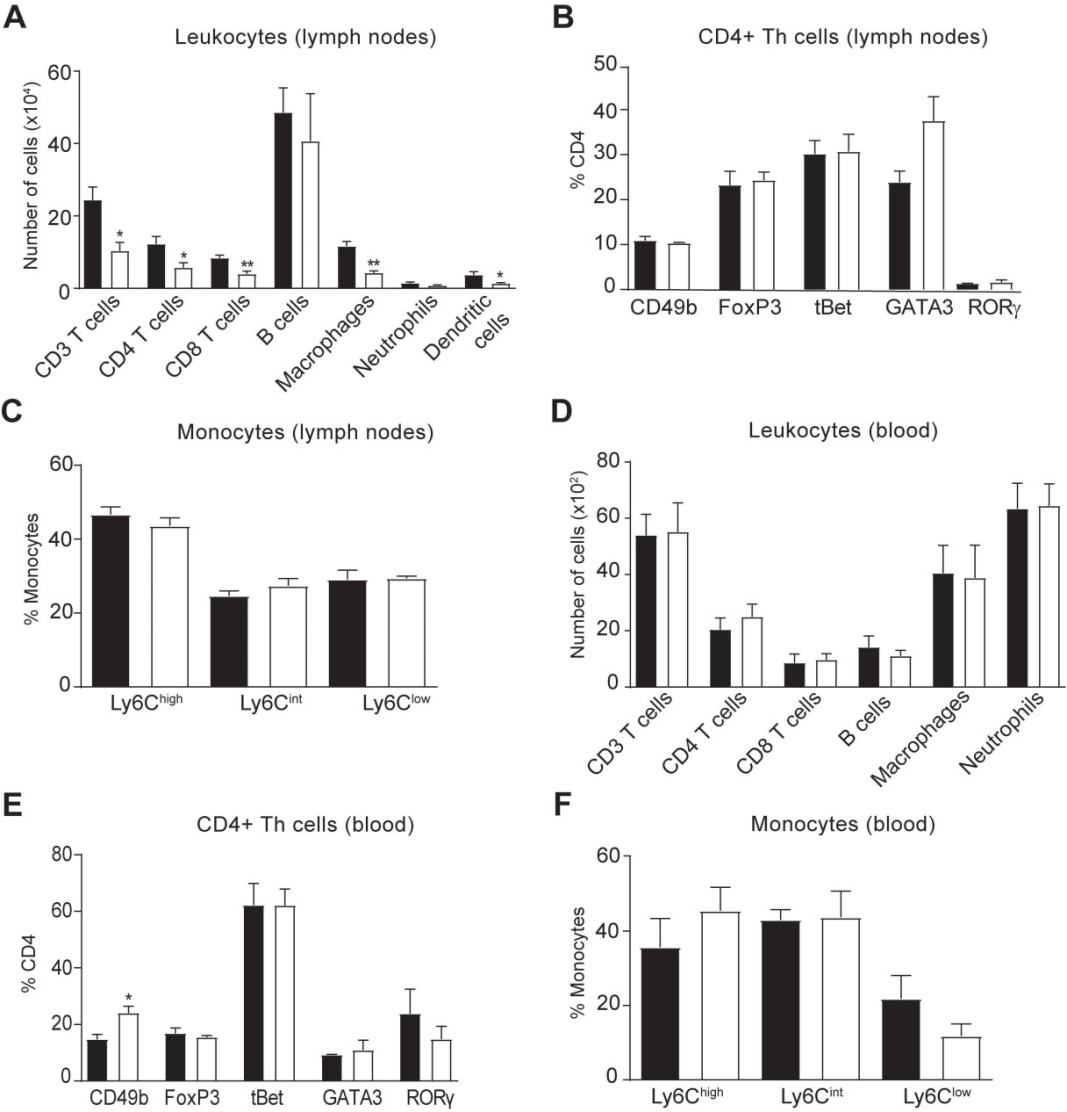
** Effects of IL-37 on immune cell counts in the lymph nodes and blood in EAE mice.** (**A**) Number of immune cells in the lymph nodes and (**E**) blood of female WT and female hIL-37tg mice at the peak of EAE. (**B**) Number of dendritic cells in the lymph nodes. (**C**) Percent of CD4+ T cells expressing CD49b, FoxP3, tBet, GATA3 or RORγ in lymph nodes and (**F**) blood. (**D**) Percentage of monocytes expressing Ly6C in lymph nodes and (**G**) blood. *p<0.05; **p<0.01; ***p<0.001 vs. WT. Unpaired *t*-test (n=4 per group). Data were obtained from a single experiment. Data shown as mean±SEM.

We then explored whether the reduced accumulation of immune cells in the spinal cord of hIL-37tg mice in EAE was due to a central or peripheral effect of this cytokine. Consequently, we first assessed the immune cell counts in the lymph nodes and blood at EAE disease peak. Similar to the spinal cord, flow cytometry analysis demonstrated that hIL-37tg mice featured a significantly reduced number of CD4 T cells (Figure 3A) but no changes in their phenotype (Figure 3B) in the lymph nodes, suggesting that IL-37 attenuated the expansion but not polarization of this T cell subset, respectively. IL-37 also reduced the number of macrophages and dendritic cells (CD45+, CD11b+, CD11c+) (Figure 3A), which is compatible with reduced T cell priming observed in the spinal cord (Figure 2B). The proportion of monocytes expressing Ly6C remained unaltered in lymph nodes (Figure 3C). In the blood samples, we found that IL-37 did not decrease the numbers of the different leukocyte populations (Figure 3D) but increased the proportion of non-classical regulatory CD4 T cells (Figure 3E) as reflected by increased CD49b expression. The proportion of monocytes expressing Ly6C remained unaltered in blood (Figure 3F).

Finally, we performed a haematopoiesis assay to discard that the reduction in the number of immune cells in the CNS and the periphery was due to a lower basal progenitor number in the bone marrow of hIL-37tg. This assay revealed that transgenic expression of IL-37 did not alter lymphocytes, monocytes and granulocytes formation in the bone marrow (Figure S4).

### IL-1R8 is critical for IL-37 to mediate beneficial effects in EAE

As reported previously, IL-37 is a dual-function cytokine that can mediate its biological effects by acting as an extracellular cytokine 30 or by translocating to the nucleus 15, 31. Here, we the sought to demonstrate the importance of the extracellular function of IL-37 in EAE.

**Figure 4:**
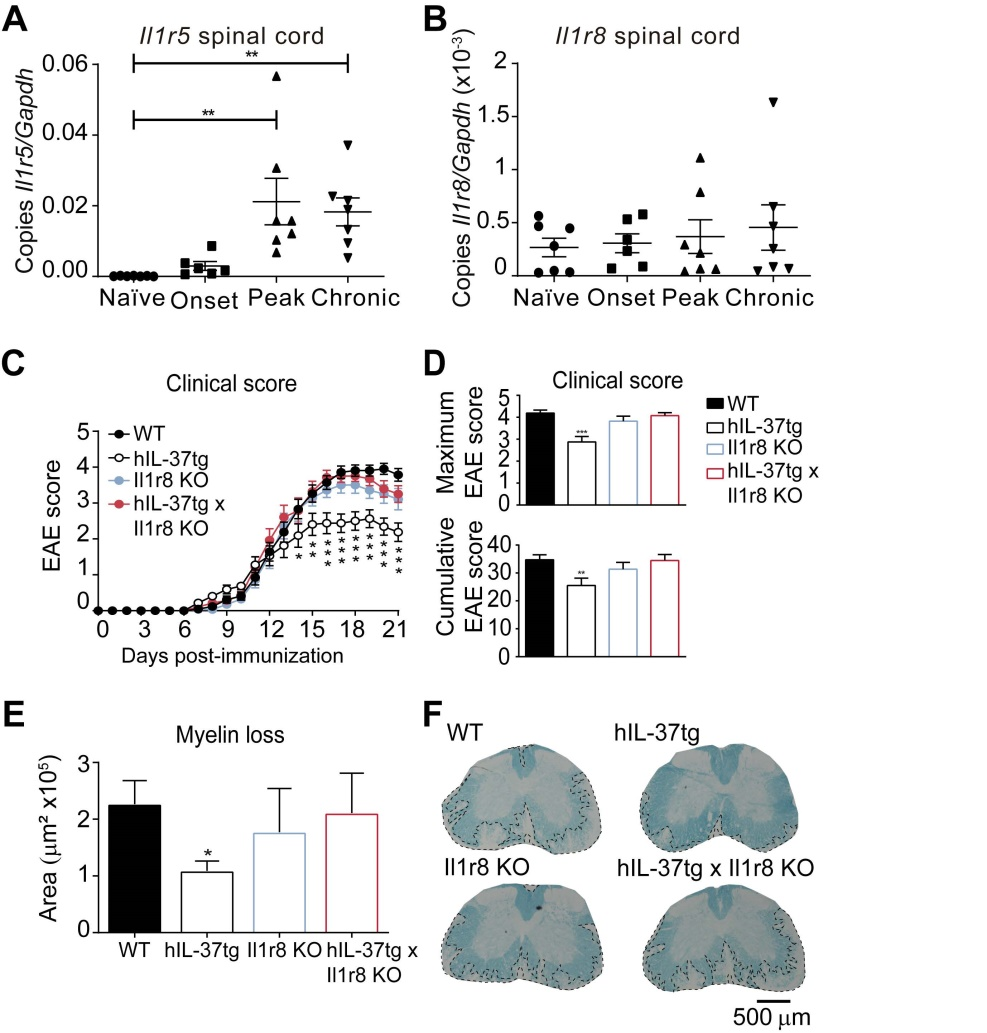
** The protective role of IL-37 expression against EAE is mediated by the IL-1R8. A-B,** Graphs showing the relative expression the *Il1r5* (A) and *Il1r8* (B) in the spinal cord of female EAE and naïve mice. (**C-D**), Clinical score of female WT, hIL-37tg, Il1r8 KO and hIL-37tg x Il1r8 KO mice showed as the mean clinical score (C), maximum and cumulative score (D). (**E**), Graph showing the quantification of myelin loss in the spinal cord of female WT, hIL-37tg, Il1r8 KO and hIL-37tg x Il1r8 KO mice at 21 days post-induction. (**F**) Representative images of the spinal cords from each of the experimental groups. *p<0.05; **p<0.01; ***p<0.001 vs. WT. One-way ANOVA in A and B (n=6 in onset and n=7 in the other groups). Two-way ANOVA with repeated measures, Bonferroni's *post hoc* test in C (n=21 in WT, n=16 in hIL-37tg, n=14, n=14 in Il1r8 KO and n=14 in hIL-37tg x Il1r8 KO. One-way ANOVA in D (n=21 in WT, n=16 in hIL-37tg, n=14 in Il1r8 KO and n=14 in hIL-37tg x Il1r8 KO. and E (n=20 in WT, n=16 in hIL-37tg, n=13 in Il1r8 KO and n=13 in hIL-37tg x Il1r8 KO. Data were pooled from two different experiments. Data shown as mean±SEM.

For this purpose, we first characterized the expression of IL-37 receptor complex in the spinal cord in different phases of EAE. We found that both components *Il1r5* and *Il1r8*, were expressed in the spinal cord at physiological conditions and during EAE disease progression (Figure 4A and 4B), although *Il1r5* transcripts significantly increased at peak and in chronic phase of EAE.

Knowing that the components of the IL-37 receptor complex were expressed in the spinal cord, we aimed at evaluating to what degree the extracellular IL-37 was important to mediate the beneficial effects of this cytokine in EAE. For this purpose, we crossed hIL-37tg mice with Il1r8 KO mice. We selected IL-1R8 for gene deletion to assess the importance of the extracellular IL-37 signalling instead of IL-1R5 because IL-1R8 is a unique co-receptor for IL-37. In contrast, IL-1R5 is also used by IL-18, and consequently, gene deletion of IL-1R5 also alters IL-18 signalling. This double transgenic mouse (*hIL-37tg x Il1r8*ko*)* expresses the human form of IL-37b in the homozygous, but this cytokine has only nuclear function, since IL-1R8, which is crucial for the extracellular function of IL-37, has been genetically deleted.

EAE induction in *Il1r8* ko mice revealed that the lack of this receptor does not alter EAE pathogenesis. However, the beneficial effects of IL-37 were completely absent in hIL-37tg mice lacking *Il1r8* (Figure 4C-D). Accordingly, histological analysis revealed that the protective actions of IL-37 against demyelination were abrogated in the absence of *Il1r8* (Figure 4E-F). These experiments provide clear evidence that the extracellular function of IL-37 is crucial in conferring protection against EAE.

### Administration of recombinant human IL-37 protein reduces neurological deficits and demyelination in EAE mice

**Figure 5:**
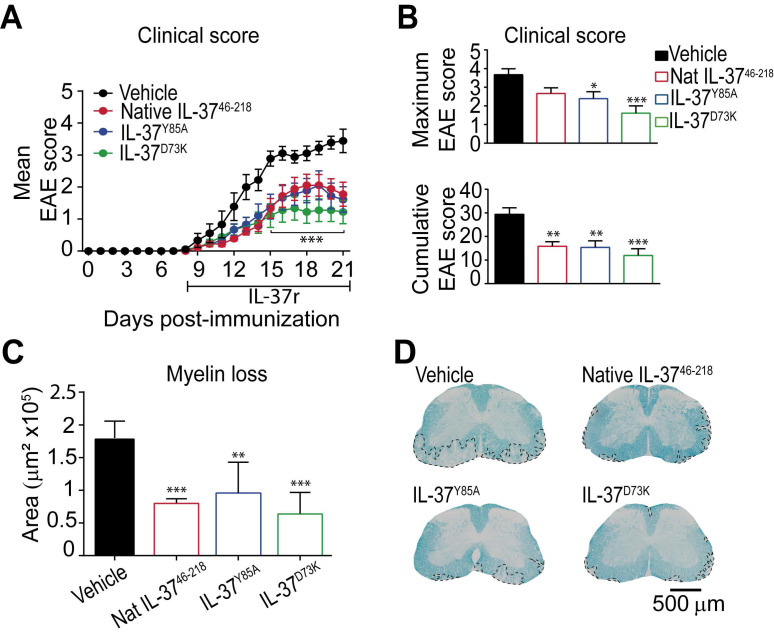
** The administration of recombinant human IL-37 protein in EAE mice.** (**A**) Clinical score of female WT EAE mice treated with IL-37^46-218^, IL-37^D73K^_,_ IL-37^Y85A^ or vehicle showed as mean clinical score and as (**B**) maximum and cumulative score. (**C**) Graph showing the quantification of demyelinating areas in the spinal cord of female WT mice treated with native IL-37^46-218^, IL-37^D73K^_,_ IL-37^Y85A^ or vehicle and (**D**) representative images of these spinal cords. *p<0.05; **p<0.01; ***p<0.001 vs. vehicle. Two-way ANOVA with repeated measures, Bonferroni's *post hoc* test in A (n= 9 per group in vehicle, IL-37^D73K^ and IL-37^Y85A^ and n=10 in native IL-37^46-218^). One-way ANOVA in B and C (n= 9 per group in vehicle, IL-37^D73K^ and IL-37^Y85A^ and n=10 in native IL-37^46-218^). Data were pooled from two different experiments Data shown as mean±SEM.

Since mice expressing IL‑37 have less disease in the EAE model, we administered recombinant human IL-37 protein in EAE mice starting at disease onset. Since IL-37 form spontaneous dimers, which have reduced anti-inflammatory actions 28, 32, we compared the efficacy of the native IL-37 (IL-37^46-218^) to two mutant monomeric forms (IL-37^D73K^ and IL-37^Y85A^), which do not dimerize. We found that daily administration of 1 µg of native IL-37^46-218^, IL-37^D73K^ or IL-37^Y85A^ from the onset of EAE enhanced functional outcomes and protected against demyelination (Figure 5A-D). Among the three recombinant IL-37 proteins, the mutant monomeric form IL-37^D73K^ showed the greatest efficacy at both functional and histological levels, although not reaching statistical significance when compared to IL-37^46-218^ and IL-37^Y85A^ (Figure 5A-D). Interestingly, we found that human recombinant IL-37^46-218^ protein led to similar therapeutic actions than Fingolimod, one of the most effective drugs to treat relapsing MS (Figure S5).

Overall, these data support the therapeutic actions of exogenous administration of recombinant human IL-37 protein in EAE.

### *IL37* is aberrantly induced in MS patients

**Figure 6:**
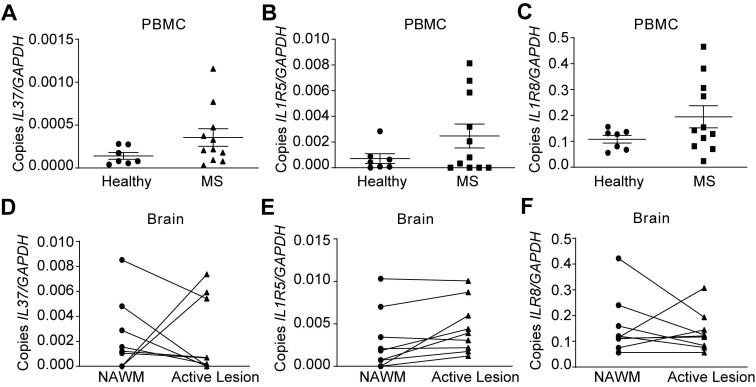
** Characterization of the expression of *IL37*, *IL1R5* and *IL1R8* in PBMC and brain from healthy and MS patients.** (**A-F**) Graphs showing the expression of IL-37 and the components of its extracellular receptor in PBMCs (A-C) and brain (D-F) from healthy and MS patients. Unpaired *t*-test in A, B and C (n=7 in healthy controls, n=11 in MS). Paired *t-*test in D, E and F (n=9 per group). Data shown as mean±SEM.

Since IL-37 plays a beneficial role in EAE, we finally evaluated IL‑37 expression in MS patients. We assessed the expression of* IL37* as well as IL‑1R5 and IL‑1R8 in PBMCs of MS patients and healthy controls. In addition, we examined brains samples from MS patients. qPCR analysis revealed that *IL37* transcripts were barely detectable in PBMC from healthy controls, and that the levels of this anti-inflammatory cytokine were not significantly increased in MS patients (Figure 6A), although its receptor components were expressed (Figure 6B-C). However, *IL37* was detectable albeit at very low levels in NAWM of MS brains. Strikingly, *IL37* levels tended to decrease in MS active lesions, except in two of the patients who showed a marked increase in the transcripts for this cytokine (Figure 6D). In line with the PBMC data, *IL1R5* and *IL1R8* were also expressed in the NAWM of brain MS samples, and the level of *IL1R5* tended to increase in active lesions (Figure 6E-F). These data reveal that while the components of the IL-37 receptors are present in the PBMC and brains of MS individuals, there appears to be a paucity of IL-37 transcripts, indicative of a relative deficiency state.

## Discussion

In the present study, we investigated whether the anti-inflammatory cytokine IL-37 protects hosts from EAE pathogenesis. We observed amelioration of the clinical signs of EAE and reduced demyelination in mice expressing human IL-37. The suppression of the inflammatory response correlates with a decrease in the protein levels of several pro-inflammatory cytokines in the spinal cord. Accordingly, IL-37 reduces the number of CD4 T cells, macrophages and dendritic cells in the lymph nodes, and attenuates the accumulation of most of the immune cell populations into the CNS. Moreover, IL-37 switches the phenotype of CD4 T cells and macrophages in the CNS resulting in more regulatory and less inflammatory phenotypes. We also observed that the beneficial actions of IL-37 are mediated by signalling via its surface receptor complex IL-1R5/IL-1R8. Lastly, we provided a proof of concept that the administration of recombinant human IL-37 protein in EAE mice alleviates disease symptomatology and protects against demyelination. Thus, we hypothesise that this natural mechanism to attenuate inflammation might be aberrant in MS patients.

IL-37 is a member of the IL-1 family that exerts potent anti-inflammatory action 33. Although this cytokine is not present in mice, the generation of a transgenic mouse that expresses the human form of IL-37, and the use of recombinant human IL-37 protein, have demonstrated its protective properties from a myriad of pathological challenges, such as colitis 34, metabolic syndrome and type 2 diabetes 18, lung inflammation 19, cancer 35, arthritis 36, calcific valve disease 37 and spinal cord injury 21.

In hIL-37tg mice, *IL37* transcription is artificially regulated by a cytomegalovirus promoter, which is constitutively expressed in all the cells 9. However, IL-37 is not constitutively expressed as expected in the hIL-37tg mouse due to the presence of an instability sequence in the mRNA that limits its half-life. Nonetheless, under inflammatory conditions, *IL37* mRNA is stabilized and, consequently, the translation of the protein is allowed 38. Along this line and similar to previous findings 21, we found that *IL37* mRNA is present at very low levels in the spinal cord of hIL-37tg mouse at steady-state conditions but is markedly upregulated during EAE. Indeed, *IL37* mRNA reached the maximum expression at peak of EAE, a disease phase when leukocytes are highly accumulated in the spinal cord, which may suggest that leukocytes are the main source of this cytokine 39. It is important to also note that *IL37* expression was associated with reduced neurological impairments and demyelination of EAE mice, supporting the beneficial action of IL-37 in neuroinflammatory conditions, as we previously demonstrated in spinal cord injury 21.

It is well established that the cytokines produced by the initial wave of CNS-infiltrating leukocytes are the dominant force that orchestrates the ensuing inflammatory cascade in MS and EAE 40, 41. These cytokines play a key role in the recruitment and activation of more immune cells from the circulation, as well as in the triggering of glial reactivity 41; they are thus key contributors to MS pathology. In support of this concept, cytokines appear among the most associated risk factor genes for MS in genome-wide association study 42.

Here, we report that transgenic expression of *IL37* silenced several pro-inflammatory cytokines in the spinal cord that have a key contribution to EAE and MS pathology 43-47. The effects of IL-37 on cytokine levels were associated with reduced infiltration of immune cells into the spinal cord at the peak severity of the disease. This is quite relevant since leukocytes accumulate in demyelinating areas and their numbers correlate to tissue damage in MS patients and EAE mice 48, 49.

Cytokines are also responsible for the polarization of immune cells, including T cells 41. The increased proportion of T regulatory cells has been defined as a hallmark of the recovery phase in EAE mice 50 as well as, of the remission phase in RRMS individuals 51. In this line, transgenic expression of *IL37* reduced the percentage of pathogenic Th1 effector cells while increasing the percent of T regulatory cells in the spinal cord at the peak of the EAE disease. Importantly, we found that the polarizing effects of IL-37 were not restricted to CD4 T cells, since hIL-37tg mice also showed lower proportion of macrophages expressing CD16/32 and iNOS, which have potent pro-inflammatory effects and contribute to EAE pathology 52. We also found decreased numbers of various immune cell populations in the lymph nodes of EAE mice with transgenic *IL37* expression. For example, there was a ~50% reduction in the number of dendritic cells, which are potent antigen-presenting cells endowed with the ability to prime T-cell responses. Both the number of MS plaques and the severity of EAE have been found to correlate with the presence and functional status of dendritic cells 53, 54. The number of CD4 T cells was also significantly reduced in the lymph nodes of hIL-37tg mice, suggesting that the expansion of this T cell subset was attenuated by IL-37. However, this cytokine did not alter the phenotype of CD4 T cells in the lymph nodes. Similarly, IL-37 did not impede the efflux of immune cells into the circulation since the number of the different immune cells analysed did not change after its transgenic expression. However, IL-37 may have favoured the mobilization of regulatory CD4 T cells into the circulation since this immune cell subset was increased in the hIL-37tg mice after EAE. These data therefore suggest that the reduced accumulation of the immune cells in the spinal cord of EAE mice with transgenic expression of *IL37* was likely due to the ability of this cytokine to reduce their infiltration into CNS rather than suppressing their mobilization into the circulation.

IL-37 is a dual-function cytokine that can exert its anti-inflammatory activity through two different pathways: (i) the activation of the extracellular receptor complex IL-1R5/IL-1R8 or (ii) its translocation into the nucleus 14. We here demonstrate that the interaction of IL-37 with its extracellular receptor complex is required to mediate its protective effects in EAE, by showing that the genetic deletion of IL‑1R8, the co-receptor for IL‑37, abolished its beneficial actions in EAE. It is important to note that this does not rule out the possibility that anti-inflammatory effects linked to the nuclear function of IL-37 likely take place in humans and may influence MS, as we recently observed in endotoxemia in mice 15.

Although IL-37 is up-regulated after inflammatory challenges there is accumulating evidence corroborating that IL-37 is deficient in some pathological conditions 37, 55-57. A recent study revealed that the production of IL-37 by T cell is reduced in MS patients. In addition, in the same study the authors demonstrate that those patients who had higher levels of IL-37 in PBMCs were more protected against to the exacerbation of the disease than those with lower levels 22. Although we did not observed reduction of *IL-37* in PBMCs of MS patients, this cytokine was not up-regulated as compared to heathy volunteers. In this line, we also observed that *IL-37* was not upregulated in brain active lesions of most MS patients. Nonetheless, the components of the extracellular receptors for IL-37 were expressed in PBMCs and brain samples of healthy donors and MS patients. These data therefore suggest that although MS individuals have the machinery to activate this beneficial mechanism to contain inflammation, IL-37 signalling is not properly induced due to low production of IL-37. Importantly, we showcase that the exogenous administration of recombinant human IL-37 protein in EAE mice ameliorated the clinical and histopathological signs of the disease, suggesting the therapeutic potential of IL-37 in MS. Highlight that treatment with recombinant IL-37 protein was initiated once mice showed the first clinical signs of EAE, which makes these findings clinically relevant.

Recent studies have reported that IL-37 forms dimers with nanomolar affinity, resulting in limited bioactivity 32. They uncovered that mutations in the dimer interface, such as IL-37^D73K^ and IL-37^Y85A^, specifically disrupt dimer formation, resulting in stable monomers that provide greater suppression of inflammation compared with native IL-37 (Eisenmesser *et al.*, 2019). However, we did not find any significant enhanced effect of the two mutant monomers IL-37^D73K^ and IL-37^Y85A^ compared to the native IL-37 form, although the monomer IL-37^D73K^ resulted in slight greater efficacy in EAE. This may indicate that the amount of IL-37 we administered was not high enough to favour the formation of dimers. Importantly, we found that human recombinant IL-37 protein exerted similar therapeutic potential than fingolimod, one of the most effective treatment for relapsing-remitting forms of MS. Interestingly, a recent study reported that serum levels of IL-37 were higher in MS individuals treated with fingolimod as compared to patients receiving no treatment, suggesting that this drug could exert its beneficial actions in MS, at least in part, by increasing the levels of this anti-inflammatory cytokine 22.

In conclusion, in the present study we provide clear evidence that IL-37 wields beneficial effects in EAE by signalling through the surface receptor complex IL-1R5/IL-1R8. Although further studies are needed to elucidate the precise role of IL-37 in MS patients, our data suggest that this cytokine could is a candidate for the treatment of MS patients.

## Supplementary Material

Supplementary figures and tables.

## Abbreviations

EAE: experimental autoimmune encephalomyelitis
IL-37: Interleukin-37
IL-1R5: interleukin 1 receptor 5
IL-1R8: interleukin 1 receptor 8
LFB: luxol fast blue
MS: multiple sclerosis
NAWM: normal appearing white matter
PBMCs: peripheral blood mononuclear cells
WT: wild type
