# Supplementary material for: IL-37 exerts therapeutic effects in experimental autoimmune encephalomyelitis through the receptor complex IL-1R5/IL-1R8
Source: Theranostics. 2021 Jan 1;11(1):1–13. doi: 10.7150/thno.47435 (PMC7681099; doi:10.7150/thno.47435)
Supplement: Supplementary file 1 — Supplementary figures and tables. [file thnov11p0001s1.pdf]

SUPLEMMENTARY FIGURES

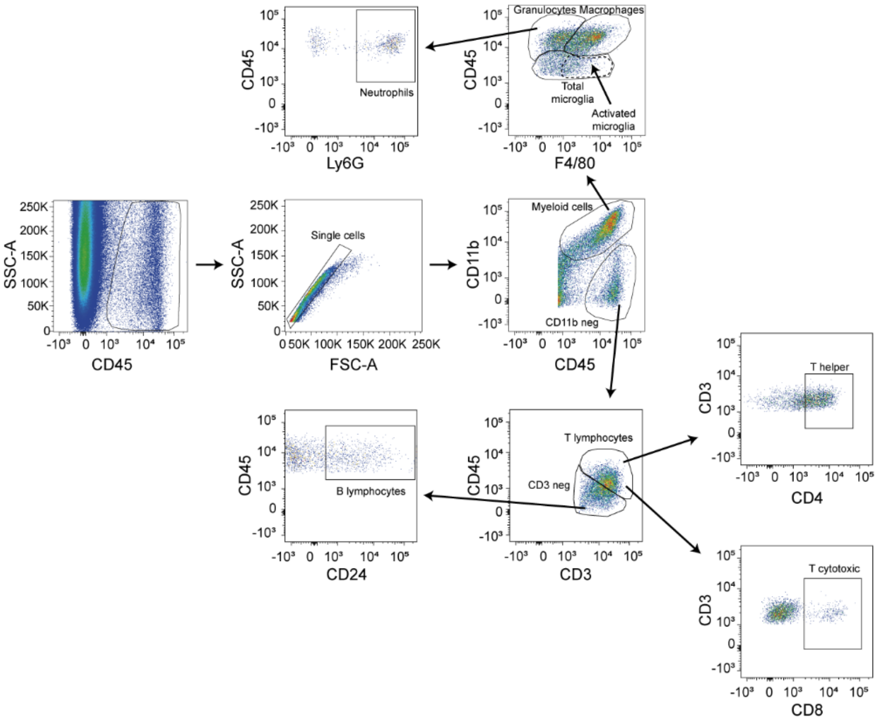

Figure S1. Representative dot plots showing the gating strategy of the main immune cell populations.

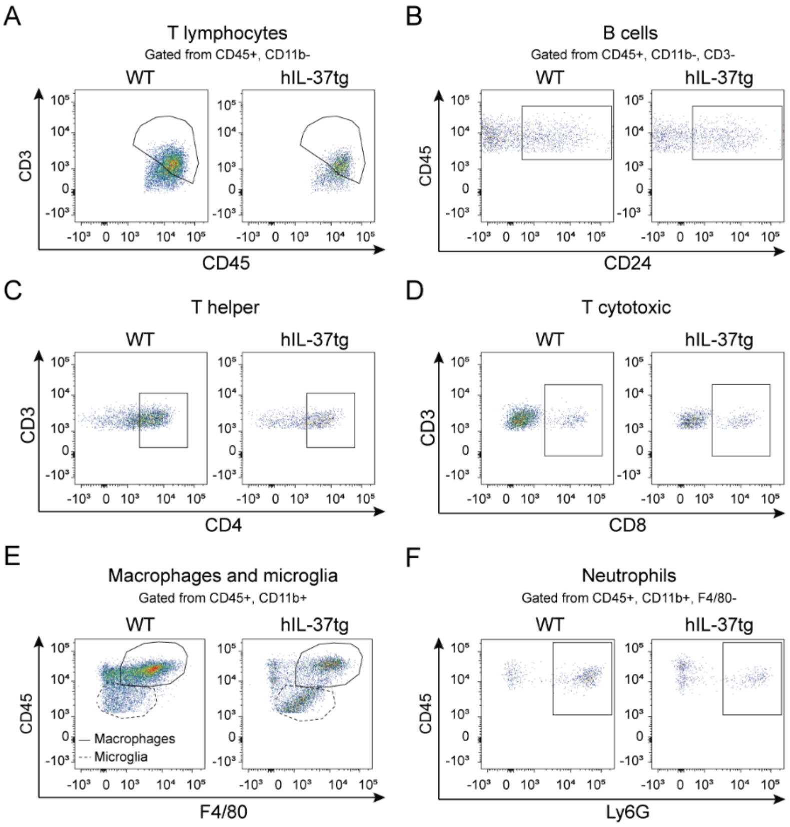

Figure S2. IL-37 reduces the accumulation of immune cells in the spinal cord of hIL-37tg mice at the peak of EAE. **(A-F)** Representative dot plots showing different **(A)** T lymphocytes, **(B)** B cells, **(C)** T helper cells, **(D)** T cytotoxic cells, **(E)** macrophages and microglia and **(F)** neutrophils in the spinal cord at the peak of EAE in WT and hIL-37tg mice.

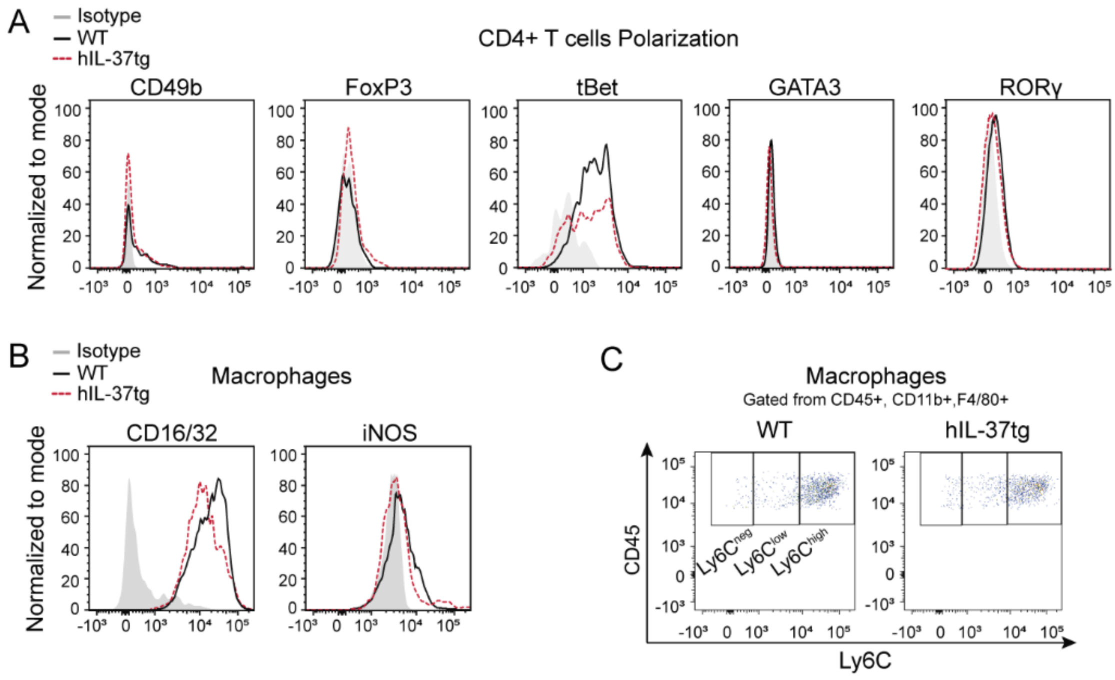

Figure S3. IL-37 modulates T cells responses in the spinal cord of hIL-37tg EAE mice. (A-C) Representative flow cytometry histograms characterising the expression of different markers of T cell responses **(A)**, pro-inflammatory markers (CD16/32 and iNOS) and **(C)** Ly6C expression in macrophages in the spinal cord at the peak of EAE in WT and hIL-37tg mice.

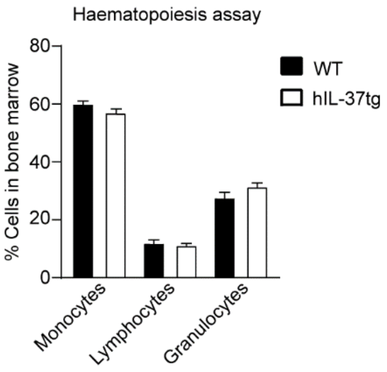

Figure S4. IL-37 does not alter the haematopoiesis in the bone marrow of hIL-37tg EAE mice. Counts of monocytes, lymphocytes and granulocytes in the bone barrow of WT and hIL-37tg. Unpaired t-test (n=4 per group). Data shown as mean±sem.

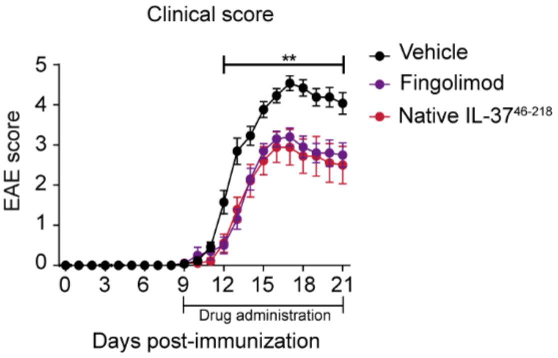

Figure S5. The administration of recombinant human IL-37 protein in EAE mice protects against EAE functional deficits in the same measure than Fingolimod. Clinical score of mice treated with vehicle, Fingolimod or native IL-$37^{46\text{-}218}$ recombinant protein showed as mean clinical score. Two-way ANOVA with repeated measures, Bonferroni's *post hoc* test.(n=13 in vehicle, n=10 in Fingolimod and n=9 in native IL-$37^{46\text{-}218}$). Data were obtained by pooling the data from two different experiments. ** $p<0.01$ vs vehicle. Data shown as mean±sem.

SUPPLEMENTARY TABLE LEGENDS

ID Sample	Type of sample	Sex	Age	Type of MS	MS duration (years)	Last relapse (years from sample collection)	EDSS	Treatment
**MS-1**	Brain	F	55	SPMS	11	–	5	Untreated
**MS-2**	Brain	F	50	SPMS	11	–	9	Untreated
**MS-3**	Brain	M	48	SPMS	6	–	8.5	Untreated
**MS-4**	Brain	F	60	SPMS	28	–	8.5	Untreated
**MS-5**	Brain	F	49	RRMS	Unknown	–	Unknown	Untreated
**MS-6**	Brain	M	26	RRMS	12	–	9	Untreated
**MS-7**	Brain	M	65	SPMS	15	–	9	Untreated
**MS-8**	Brain	M	61	SPMS	26	–	9.5	Untreated
**MS-9**	Brain	F	44	SPMS	Unknown	–	Unknown	Untreated
**MS-10**	PBMCs	F	63	RRMS	1	11	1	Untreated
**MS-11**	PBMCs	F	45	RRMS	16	9	1	Untreated
**MS-12**	PBMCs	F	36	RRMS	2	3	0	Untreated
**MS-13**	PBMCs	M	65	SPMS	22	22	6.5	Untreated
**MS-14**	PBMCs	M	31	RRMS	1	1	1	Copaxone
**MS-15**	PBMCs	F	45	SPMS	16	5	11	Untreated
**MS-16**	PBMCs	F	44	RRMS	18	18	0	Untreated
**MS-17**	PBMCs	F	60	SPMS	14	11	8	Corticosteroids
**MS-18**	PBMCs	F	34	RRMS	5	5	0	Untreated
**MS-19**	PBMCs	M	27	RRMS	1	0	0	Untreated
**MS-20**	PBMCs	F	27	RRMS	6	5	0	Untreated
**HC-1**	PBMCs	M	25	Healthy	–	–	–	–
**HC-2**	PBMCs	F	26	Healthy	–	–	–	–
**HC-3**	PBMCs	M	37	Healthy	–	–	–	–
**HC-4**	PBMCs	F	30	Healthy	–	–	–	–
**HC-5**	PBMCs	F	57	Healthy	–	–	–	–
**HC-6**	PBMCs	M	27	Healthy	–	–	–	–
**HC-7**	PBMCs	F	42	Healthy	–	–	–	–

Supplementary Table 1. Descriptive information about human samples of MS patients and healthy controls.

	Naive WT		Naive hIL-37tg		t-test	WT vs. hIL-37tg
	(pg ck/mg prot)	SEM	(pg ck/mg prot)	SEM		
**IL-4**	4,99	1,05	5,37	1,56	ns	=
**IL-10**	5,85	3,42	4,13	0,86	ns	=
**IL-1α**	6,75	2,31	6,72	1,32	ns	=
**IL-1β**	0,4	0,2	0,22	0,08	ns	=
**IL-3**	0,44	0,13	0,39	0,067	ns	=
**IL-6**	5,6	0,76	15,35	4,22	ns	=
**IL-17A**	11,68	1,7	6,81	0,98	ns	=
**IFNϒ**	1,73	0,42	1,36	0,34	ns	=
**TNFα**	0	0	0,35	0,35	ns	=
**CXCL-2**	14,15	3,79	13,12	2,66	ns	=
**CXCL-10**	5,4	1,61	6,57	0,87	ns	=
**CCL-2**	19,34	4,59	11,59	1,65	ns	=
**CCL-5**	2,43	1,07	4,24	1,93	ns	=
**G-CSF**	2,82	0,38	1,3	0,14	ns	=
**GM-CSF**	0	0	0	0	ns	=

	EAE 3 days post-onset WT		EAE 3 days post-onset hIL-37tg		t-test	WT vs. hIL-37tg
	(pg ck/mg prot)	SEM	(pg ck/mg prot)	SEM		
**IL-4**	2,62	0,29	2,15	0,17	ns	=
**IL-10**	208,58	47,68	192,99	64,01	ns	=
**IL-1α**	10,57	2,37	2,74	0,59	*	↓
**IL-1β**	3,27	0,58	0,93	0,49	***	↓
**IL-3**	0,06	0,06	0,05	0,03	ns	=
**IL-6**	21,27	3,84	5,41	1,18	**	↓
**IL-17A**	5,86	0,74	5,2	1,42	ns	=
**IFNϒ**	0,88	0,06	0,34	0,06	**	↓
**TNFα**	2,15	0,49	0,86	0,07	*	↓
**CXCL-2**	18,6	3,3	15,85	3,2	ns	=
**CXCL-10**	208,58	47,68	192,99	64,01	ns	=
**CCL-2**	37,13	11,16	34	10,52	ns	=
**CCL-5**	12,42	3,97	9,03	4,17	ns	=
**G-CSF**	4,3	0,3	4,2	0,4	ns	=
**GM-CSF**	0,79	0,13	0,8	0,06	ns	=

Supplementary Table 2. Cytokine expression in the spinal cord of WT and hIL-37tg mice at physiological conditions and at 3 days after EAE onset. *$p<0.05$; **$p<0.01$; ***$p<0.001$ vs. WT. Unpaired t test was used to analyze significant differences between groups (n=4 per group in naïve mice; n=5 per group in EAE mice). Data is shown as mean±SEM.
